# Large eddy simulation on wind-induced interference effects of staggered chamfered square cylinders

**DOI:** 10.1038/s41598-023-44711-5

**Published:** 2023-10-16

**Authors:** Wenlong Cao, Xueyan Wang, Yubo Liu, Yujie Yin, Jian Yang, Jianhui An

**Affiliations:** 1 Department of Road and Bridge Engineering, Hebei Jiaotong Vocational and Technical College, Shijiazhuang, 050091 China; 2 School of Economics and Management, Shijiazhuang University, Shijiazhuang, 050091 China; 3 Hebei Construction Group Co., Ltd., Shijiazhuang, 050051 China

**Keywords:** Civil engineering, Environmental impact, Computational science

## Abstract

Chamfered corners in buildings are the main means to reduce the control effect of wind load on the structure, and the interference effect of chamfered buildings cannot be ignored. At present, only the mutual interference coefficients of square and rectangular section buildings are given in the Chinese code, without the interference effect of chamfered buildings being specified. Therefore, in this paper, aerodynamic force and wind pressure coefficients of chamfered square cylinders of different spacing are obtained by the large eddy simulation method. Wind load characteristics, non-Gaussian characteristics and interference effects of chamfered square cylinders with different arrangements are studied based on aerodynamic coefficients, wind pressure coefficients and interference coefficients. The results show that when the wall y plus value is 1, the large eddy simulation is the most accurate to simulate the wind load and wind field parameters. Besides, the aerodynamic effects, non-Gaussian characteristics and interference effects between the chamfered square cylinders are mainly controlled by the cross-wind interval and the spacing (4.0, 4.0) is the characteristic coordinate. That means, when the spacing is smaller than this coordinate, the interference effect of the square cylinder is more obvious. When the spacing coordinate is greater than (4.0, 4.0), the aerodynamic coefficients and non-Gaussian regional distributions of the principal square cylinder and the isolated cylinder are the same, and the interference factor approaches 1.

## Introduction

Unlike traditional low-rise buildings, with the growth of height the lateral stiffness of high-rise buildings decreases and flexibility increases. At the same time, before reaching gradient wind height, the wind speed increases with the height exponentially or logarithmically. As a result, for high-rise buildings, the wind load often plays a controlling role. For this reason, in high-rise buildings, aerodynamic measures are usually adopted to reduce wind load by changing the shape of buildings. For example, Taipei 101 just uses a rounded design, which reduces the base bending moment of the structure by 25%^1^. What’s more, Shanghai Center also introduces such aerodynamic optimizations as three-dimensional curved surfaces and rotation patterns, reducing the wind load of the structure by 60%^2^. Reasonable aerodynamic measures of shape design can not only meet the aesthetic requirements of buildings, but also matter significantly for safety, applicability, and economics.

Chamfered treatment which is easy to operate and cost saving is one of the more effective aerodynamic optimization measures mainly by changing the building shape to meet the requirement of wind drag or be more wind-proof^3–5^. At present, the main treated methods include rounded corners, concave corners, chamfered corners, etc., among which chamfered corners are more commonly seen. Studies have shown that a 10% chamfered rate can significantly reduce the cross-wind aerodynamic force of the structure and avoid aerodynamic instability^6–9^. Under the precondition of ensuring the same area of use (the reduced area of the corner treatment being compensated by increasing the height), the wind vibration response of the chamfered high-rise building is still smaller than that of the untreated building^10^.

At present, the research on chamfered buildings is mainly limited to isolated buildings, but in reality, high-rise buildings often appear in groups, thus the interference effect among chamfered high-rise buildings cannot be ignored. However, in the Load Code for the Design of Building Structures GB50009-2012^11^, only the interference coefficients of buildings with same-height rectangular sections are clearly specified, and there is no quantitative conclusion on the interference coefficients among other sections. In the current research on the group interference effect, standard square cylinders without being corner-treated are often taken as the research object^12–16^. Therefore, it is very necessary to study the interference effect of chamfered high-rise buildings, which can be highly referred to in engineering applications.

In this paper, large eddy simulation is used to numerically simulate chamfered square cylinders with the arbitrary arrangement in a uniform flow field, and aerodynamic and wind pressure time history under 120 working conditions at different coordinate positions are obtained. Based on this, the aerodynamic interference effect, non-Gaussian characteristics and distribution patterns of wind-induced interference effect of chamfered square cylinders at different intervals are summarized. By comparing the interference factors of the corresponding facades in various working conditions, the most unfavorable coordinates of the interference effects of chamfered square cylinders are analyzed, and the distribution of interference factors of different facades is finally given. The materials and methods section should contain sufficient detail so that all procedures can be repeated. It may be divided into headed subsections if several methods are described.

## Governing equations of numerical simulation

The basic idea of large eddy simulation is to directly solve large-scale vortices in the flow field through a filter function, while small-scale vortices are not directly solved but calculated by introducing a sub-grid scale model. This method is not only more accurate than Reynolds Averaged Navier-Stockes, but also more efficient than direct numerical simulation which explains why this paper uses large eddy simulation based on the spatial average.

### Mass conservation equations

The mass conservation means that in unit time, the increase of fluid mass in a micro-unit is equal to the inflow of mass, and the equation is as follows^17–19^:

1 $$ \frac{\partial \rho }{{\partial t}} + \frac{\partial (\rho u)}{{\partial x}} + \frac{\partial (\rho v)}{{\partial y}} + \frac{\partial (\rho w)}{{\partial z}} = 0, $$

where *ρ* is the air density at 15 °C; *t* stands for time; *u*, *v*, and *w* represent the components of the velocity in the *x*, *y*, and *z* directions.

In this study, the air is regarded as an incompressible fluid, that is, *ρ* is a constant, and then Eq. (1) can be simplified to:

2 $$ \frac{\partial u}{{\partial x}} + \frac{\partial v}{{\partial y}} + \frac{\partial w}{{\partial z}} = 0. $$

### Momentum conservation equations

The momentum conservation equation refers to that in a micro-unit the change ratio of the momentum of the fluid to time is equal to the sum of all external forces acting on the micro-unit. The momentum conservation equation can be expressed as^20–22^:

3 $$ \begin{aligned} \frac{\partial \rho u}{{\partial t}} + {\text{div}}\left( {\rho uu} \right)\, & = - \frac{\partial p}{{\partial x}} + \frac{{\partial \tau_{xx} }}{\partial x} + \frac{{\partial \tau_{yx} }}{\partial y} + \frac{{\partial \tau_{zx} }}{\partial z} + F_{x} , \\ \frac{\partial \rho v}{{\partial t}} + {\text{div}}\left( {\rho vu} \right)\, & = - \frac{\partial p}{{\partial y}} + \frac{{\partial \tau_{xy} }}{\partial x} + \frac{{\partial \tau_{yy} }}{\partial y} + \frac{{\partial \tau_{zy} }}{\partial z} + F_{y} , \\ \frac{\partial \rho w}{{\partial t}} + {\text{div}}\left( {\rho wu} \right)\, & = - \frac{\partial p}{{\partial z}} + \frac{{\partial \tau_{xz} }}{\partial x} + \frac{{\partial \tau_{yz} }}{\partial y} + \frac{{\partial \tau_{zz} }}{\partial z} + F_{z} , \\ \end{aligned} $$

where *p* is the pressure on the micro-unit; *τ*_*xx*_, *τ*_*xy*_, *τ*_*xz*_ refer to the component of viscous stress *τ*; *F*_*x*_, *F*_*y*_, *F*_*z*_ are the physical forces on the micro body.

If the physical force *F* is ignored, Eq. (3) can be expressed as:

4 $$ \begin{aligned} \frac{\partial \rho u}{{\partial t}} + {\text{div}}\left( {\rho uu} \right)\, & = - \frac{\partial p}{{\partial x}} + \frac{{\partial \tau_{xx} }}{\partial x} + \frac{{\partial \tau_{yx} }}{\partial y} + \frac{{\partial \tau_{zx} }}{\partial z}, \\ \frac{\partial \rho v}{{\partial t}} + {\text{div}}\left( {\rho vu} \right)\, & = - \frac{\partial p}{{\partial y}} + \frac{{\partial \tau_{xy} }}{\partial x} + \frac{{\partial \tau_{yy} }}{\partial y} + \frac{{\partial \tau_{zy} }}{\partial z}, \\ \frac{\partial \rho w}{{\partial t}} + {\text{div}}\left( {\rho wu} \right)\, & = - \frac{\partial p}{{\partial z}} + \frac{{\partial \tau_{xz} }}{\partial x} + \frac{{\partial \tau_{yz} }}{\partial y} + \frac{{\partial \tau_{zz} }}{\partial z}. \\ \end{aligned} $$

### Governing equations of large eddy simulation

The mass conservation equations and momentum conservation equations in the instantaneous state are filtered, and the governing equations of large eddy simulation are obtained as^20–22^:

5 $$ \frac{\partial \rho }{{\partial t}} + \frac{{\partial \rho \overline{u}_{i} }}{{\partial x_{i} }} = 0 $$

6 $$ \frac{{\partial \rho \overline{u}_{i} }}{\partial t} + \frac{{\partial \rho \overline{u}_{i} \overline{u}_{j} }}{{\partial x_{j} }} = - \frac{{\partial \overline{p} }}{{\partial x_{i} }} + \nu \frac{{\partial^{2} \overline{u}_{i} }}{{\partial x_{i} \partial x_{j} }} - \frac{{\partial {(}\tau_{ij} {)}}}{{\partial x_{j} }}, $$

where $\overline{u}_{i}$ and $\overline{u}_{j}$ represent the filtered speed in three directions, *i, j* = 1, 2, 3; $\overline{p}$ refers to the filtered pressure; *τ*_*ij*_ is sub-grid scale stress.

The sub-grid scale stress reflects the influence of unsolved small-scale vortices on the turbulence motion of large-scale vortices, and its closed scheme must be constructed in order to solve the governing equation, so the sub-grid scale model is produced.

### Sub-grid scale model

According to the basic model proposed by Smagorinsky^23^, the sub-grid scale stress is obtained by Eq. (7), where *μ*_*t*_ is the turbulent viscosity, and it can be calculated by Eqs. (8) and (9).

7 $$ \tau_{ij} - \frac{1}{3}\tau_{kk} \delta_{ij} = - 2\mu_{t} \overline{S}_{ij} $$

8 $$ \mu_{t} = \left( {C_{s} \Delta } \right)^{2} \left| {\overline{S} } \right| $$

9 $$ \overline{S}_{ij} = \frac{1}{2}\left( {\frac{{\partial \overline{{u_{i} }} }}{{\partial x_{j} }} + \frac{{\partial \overline{{u_{j} }} }}{{\partial x_{i} }}} \right)\begin{array}{*{20}c} , & {} \\ \end{array} \left| {\overline{S} } \right| = \sqrt {2\overline{S}_{ij} \overline{S}_{ij} } \begin{array}{*{20}c} , & {} \\ \end{array} \Delta = \left( {\Delta_{x} \Delta_{y} \Delta_{z} } \right)^{1/3} , $$

where $\Delta_{i}$ represents the grid size along the *i*-axis direction; *C*_*s*_ is the Smagorinsky constant equal to 0.1 in the standard model. In numerical simulation calculation, the flow field is often complicated, and the fixed *C*_*s*_ value cannot accurately reflect the flow field state. Therefore, Dynamic Smagorinsky model is used in this paper to better consider the flow field around the blunt body of the impulse impingement, separation, free shear layer, vortex shedding and other phenomena, and a certain value of dynamic *C*_*s*_ between 0 and 0.23 is determined^24,25^.

## Model and working conditions

### Numerical model and parameter setting

Flow field: In order to minimize the effect of Reynolds number and to correspond to the references, the flow field in this paper adopts a uniform flow field with the Reynolds number of 22,000^26–29^. The incoming flow velocity is calculated to be 3.14 m/s.

**Figure 1:**
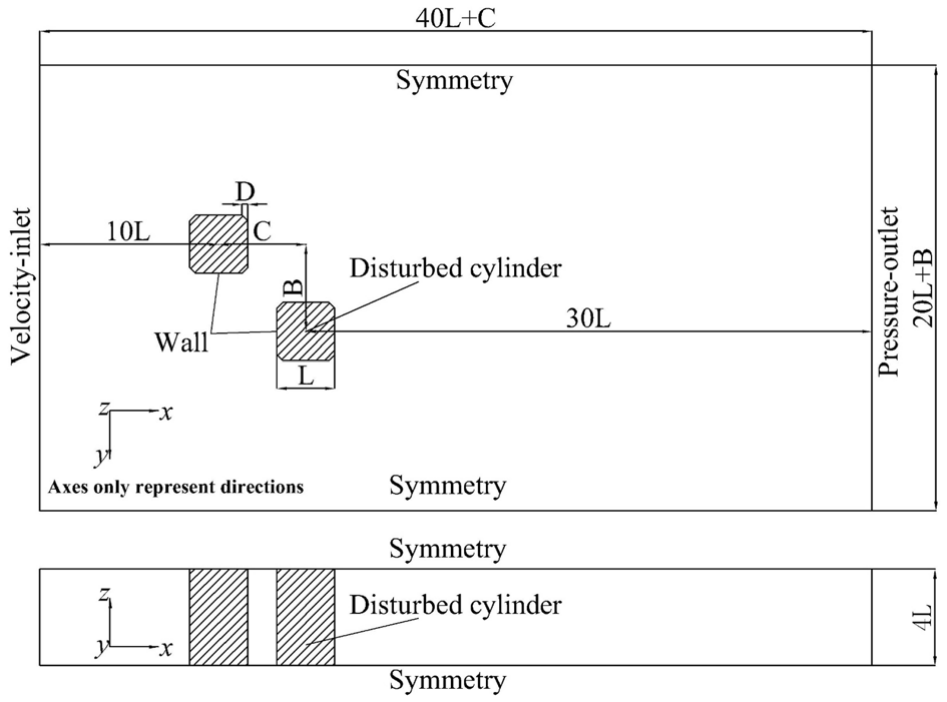
Computational domain size.

**Figure 2:**
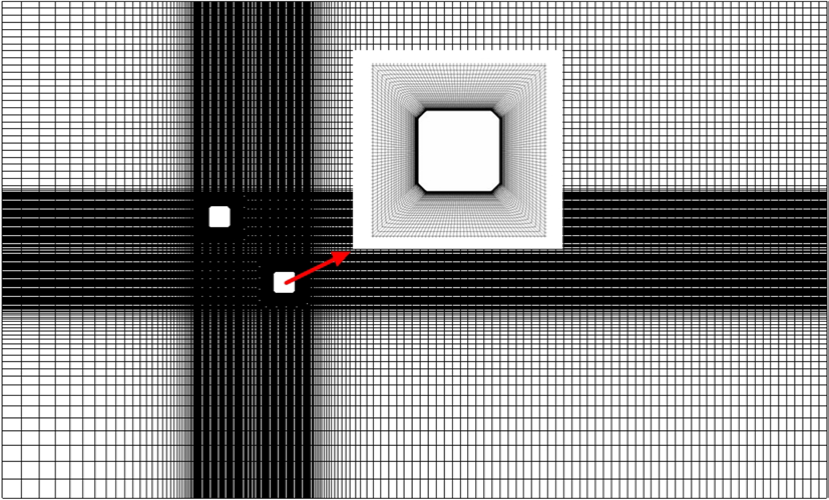
Mesh generation.

Calculation domain and grid division: the size of the calculation domain is shown in Fig. 1 in which the reference side length (L) of the square cylinder is 0.1 m, the height is 4L, the side length of the chamfered corner (D) is *λ*L, and *λ* is a constant equal to 0.1. The size of the calculation domain is (40L + C) (flow direction *x*) × (20L + B) (span direction *y*) × 4L (vertical *z*)^27,28^ to ensure that the blockage ratio is less than 5%^30^. The transverse center distance of the two square cylinders is B, and the flow center distance of the two square cylinders is C. The grid is a non-uniform hexahedron, and the near-wall grid is encrypted as shown in Fig. 2. The height of the boundary layer is 5 × 10^−5^ m, and the total number of grids is controlled to about 3 million.

Boundary conditions: the domain inlet is a Velocity-inlet; The outlet is a Pressure-outlet; On the upper and lower surfaces and both sides, Symmetry is used to simulate the free sliding wall. The surface of the square cylinder adopts a non-slip Wall.

Solution method: The solution method is SIMPLEC in which the momentum equation is of second-order precision, the convergence residual control is 5 × 10^–4^, and the time step is 5 × 10^−4^ s.

### Working condition arrangement

**Figure 3:**
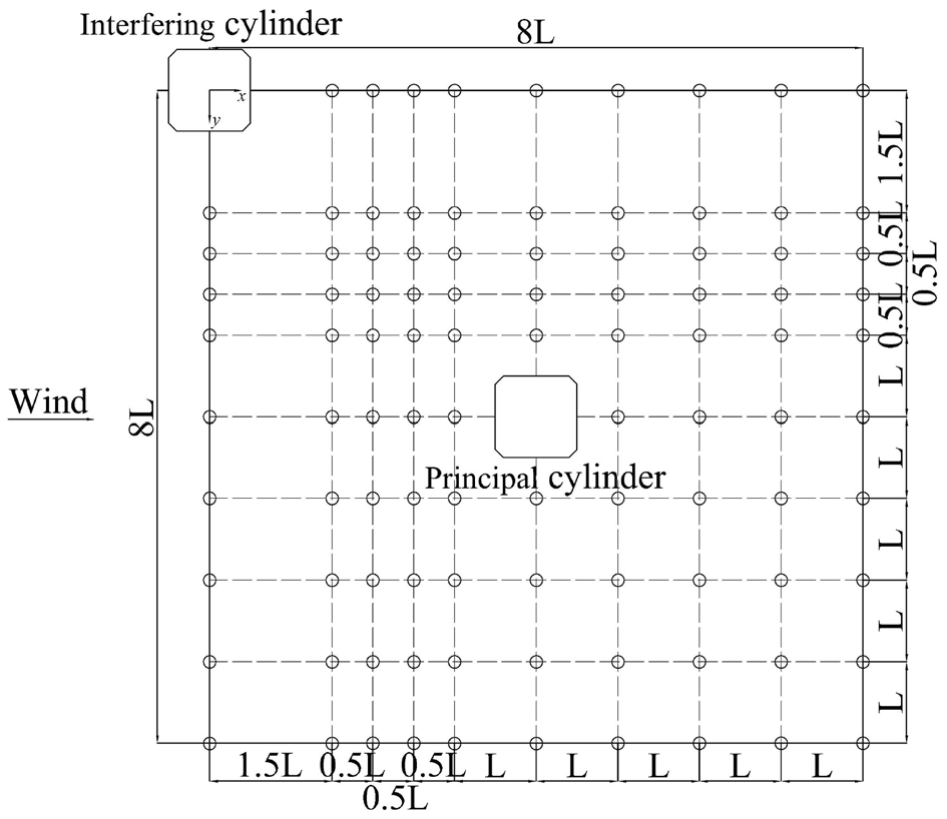
Coordinate grid system of simulation.

Set the coordinate system as shown in Fig. 3^31,32^. For the convenience of expression, the center opposition of the interfering square cylinder is defined as the origin of the coordinates, and horizontal and vertical coordinates are all normalized by L. The *x*-axis and *y*-axis directions are shown in Fig. 3, and the position of the principal cylinder is defined by coordinates, arranged in about 100 positions from 8L behind the interfering cylinder to 8L on the flank side. In the expression, the along-wind distance (*x* direction) and the cross-wind distance (*y* direction) are often used to represent the distance between two cylinders. For example, the maximum distance between two cylinders can be expressed as the along-wind distance of 8L and the cross-wind distance of 8L, namely, the coordinates are (8.0,8.0).

### Simulation verification

The accuracy and efficiency of the numerical simulation are directly related to the density of the grid in the calculation domain. In order to clarify the influence of grid density on the simulation results, four grid forms, namely, Case1 ~ Case4, are used to analyze the convergence of the grid and verify the simulation method by taking the flow around a single square cylinder as an example. The wind pressure *P* of the measuring point, model drag force *F*_*D*_ and the model lift force *F*_*L*_ presented in this paper are all dimensionless processed as^33^:

10 $$ C_{p} = \frac{{P - P_{0} }}{{1/2\rho U_{0}^{2} }} $$

11 $$ \overline{C}_{p} = \frac{{\overline{P} - P_{0} }}{{1/2\rho U_{0}^{2} }} $$

12 $$ C_{{_{P} }}^{\prime } = \frac{{P^{\prime } - P_{0} }}{{1/2\rho U_{0}^{2} }} $$

13 $$ C_{D} = \frac{{F_{D} }}{{1/2\rho U_{0}^{2} LH}} $$

14 $$ \overline{C}_{D} = \frac{{\overline{F}_{D} }}{{1/2\rho U_{0}^{2} LH}} $$

15 $$ C_{L} = \frac{{F_{L} }}{{1/2\rho U_{0}^{2} LH}} $$

16 $$ C_{L}{\prime} = \frac{{F_{L}{\prime} }}{{1/2\rho U_{0}^{2} LH}} ,$$

where *P*_0_ is the static pressure at the reference point; $\overline{P}$ and* P*^*’*^ are the mean and fluctuating values of* P*; $\overline{F}$_*D*_ is the mean value of *F*_*D*_, and* F*^*’*^_*L*_ is the fluctuating value of *F*_*L*_; *U*_0_ is the incoming wind speed; *H* is the reference height of the square cylinder; *C*_*P*_, $\overline{C}_{P}$ and *C*^*’*^_*P*_ are respectively wind pressure coefficient, average wind pressure coefficient, and fluctuating wind pressure coefficient; *C*_*D*_ and $\overline{C}_{D}$ denote the drag coefficient and average drag coefficient; *C*_*L*_ and *C*^*’*^_*L*_ represent lift coefficient and fluctuating lift coefficient.

Table 1 shows the comparison results of $\overline{C}_{D}$, *C*^*’*^_*L*_ and *S*_*t*_ under four grid schemes with those appearing in the literature review. In Table 1, *S*_*t*_ is the Strouhal number, which is a dimensionless physical quantity defined as Eq. (17), and the value of *y*^+^ represents the dimensionless distance of the grid of the first layer of the boundary layer, namely, Eq. (18).

17 $$ S_{t} = fL/V $$

18 $$ y^{ + } = \mu y/\nu , $$

where *f* is the vortex shedding frequency; *V* is the fluid velocity; *μ* is the friction velocity and *y* is the near-wall grid size.

**Table 1:** Analysis of grid convergence and comparison of results.

Program	First layer grid size	Wall *y*^+^	Number of grids	$\overline{C}_{D}$	$C_{{\text{L}}}{^\prime}$	$S_{t}$
Case1	1 × 10^−2^L	10	5.6 × 10^5^	2.058	0.842	0.132
Case2	1 × 10^−3^L	2.5	9.5 × 10^5^	2.272	1.339	0.130
Case3	5 × 10^−4^L	1	1.4 × 10^6^	2.269	1.228	0.131
Case4	1 × 10^−4^L	0.25	1.8 × 10^6^	2.372	1.365	0.132
Lee^1^	–	–	–	2.200	1.200	0.120
Reinhold et al.^2^	–	–	–	2.190	1.070	0.130
Sakamoto et al.^31^	–	–	–	2.380	1.230	0.130
Noda & Nakayama^32^	–	–	–	2.164	1.180	0.131
Alam et al.^33^	–	–	–	2.150	1.180	0.128

The *S*_*t*_ of the four grid schemes is 0.130 ~ 0.132, which is the same as the wind tunnel test results in Refs.^1,2,31–33^. The average drag coefficient and fluctuating lift coefficient of Case1 are smaller, with the fluctuating lift coefficient only 0.842, which obviously does not meet the requirement of numerical simulation accuracy. The average drag coefficients of Case2 ~ Case4 differ less than 5% from the literature review, but the fluctuating lift coefficients of Case2 and Case4 are both greater than 1.3, while the fluctuating lift coefficients of Case3 are same as the results obtained by Sakamoto et al.^31^. This result shows that in large eddy simulation, the calculation accuracy is highest when the wall *y* plus is controlled to about 1, and too many or too few grids will affect the numerical simulation results. Finally, considering the calculation accuracy and efficiency, Case3 grid scheme is adopted for the subsequent numerical calculation in this paper.

**Figure 4:**
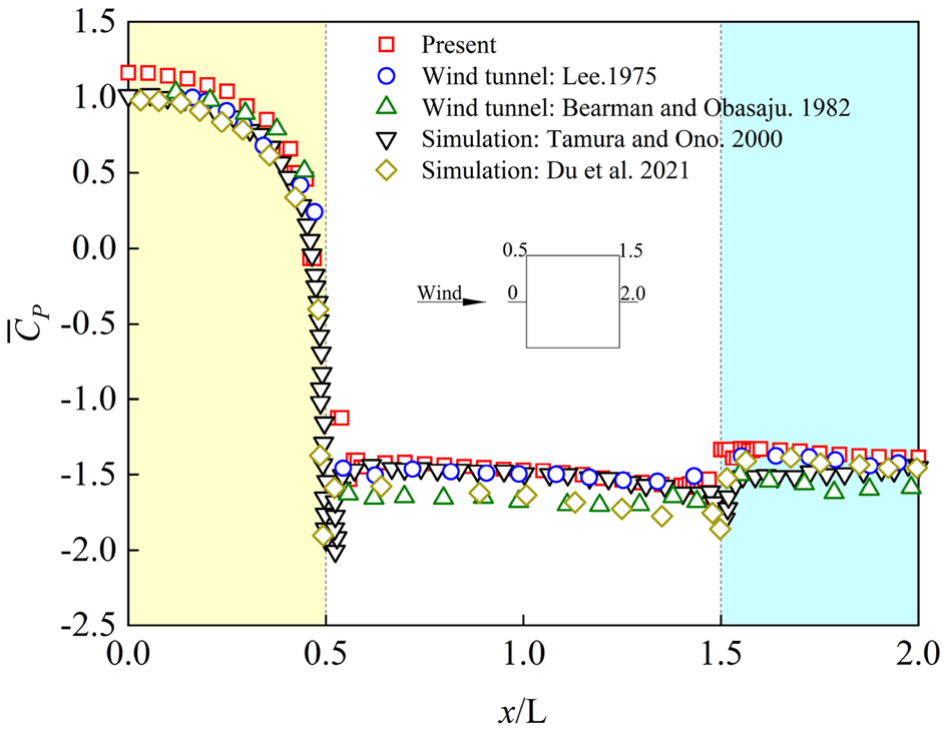
Average wind pressure coefficients.

**Figure 5:**
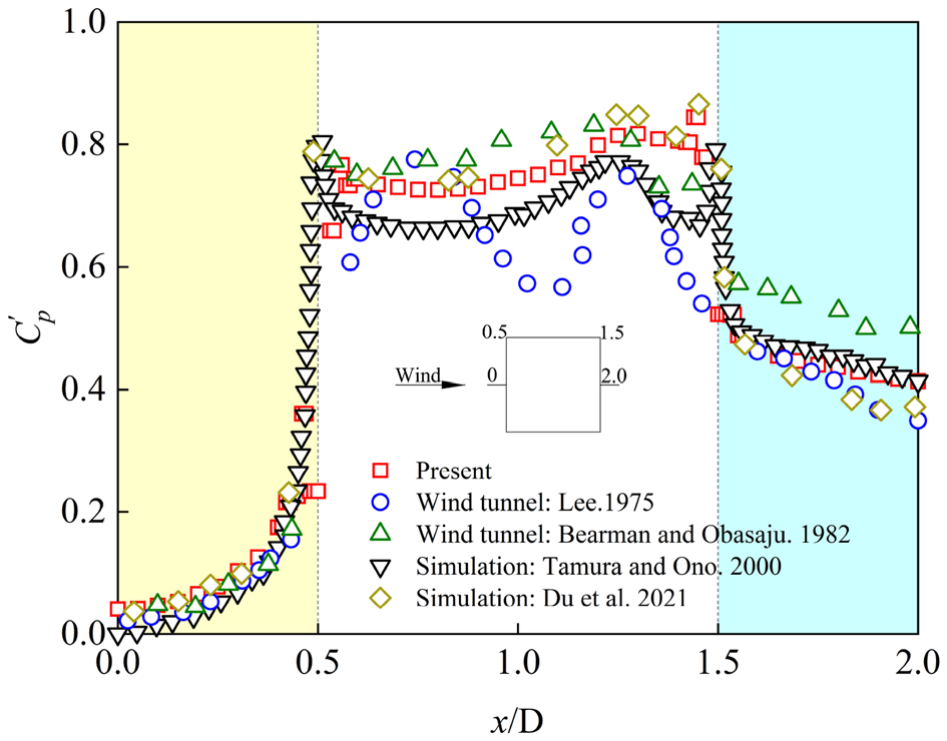
Fluctuating wind pressure coefficients.

In order to reduce the occasional comparison results, the average wind pressure coefficients and fluctuating wind pressure coefficients at the center line of the model height direction in Case3 were selected and compared with the wind tunnel test results and numerical simulation results in literature review, as shown in Figs. 4 and 5. The tendency and value of average wind pressure coefficients and fluctuating wind pressure coefficients in numerical simulation is consistent with what is obtained in Refs.^1,34–37^. However, the fluctuating wind pressure coefficients of the side elevation of the square cylinder (coordinate corresponding to 0.5 ~ 1.5) is larger than the wind tunnel test value of Lee^1^, but the results are similar to those of Berman and Obasaju^34^. The reason for this result may be that compared with numerical simulation, wind tunnel test has more interference from external factors, such as transmission error of pipelines and turbulence of incoming wind field. Based on the above comparison results, the numerical simulation method selected in this paper can accurately reflect all items of aerodynamic values and wind pressure results of the square cylinder, and can obtain more accurate and reliable results in the subsequent numerical calculation.

## Results and discussions

### Wind characteristics of chamfered column

**Figure 6:**
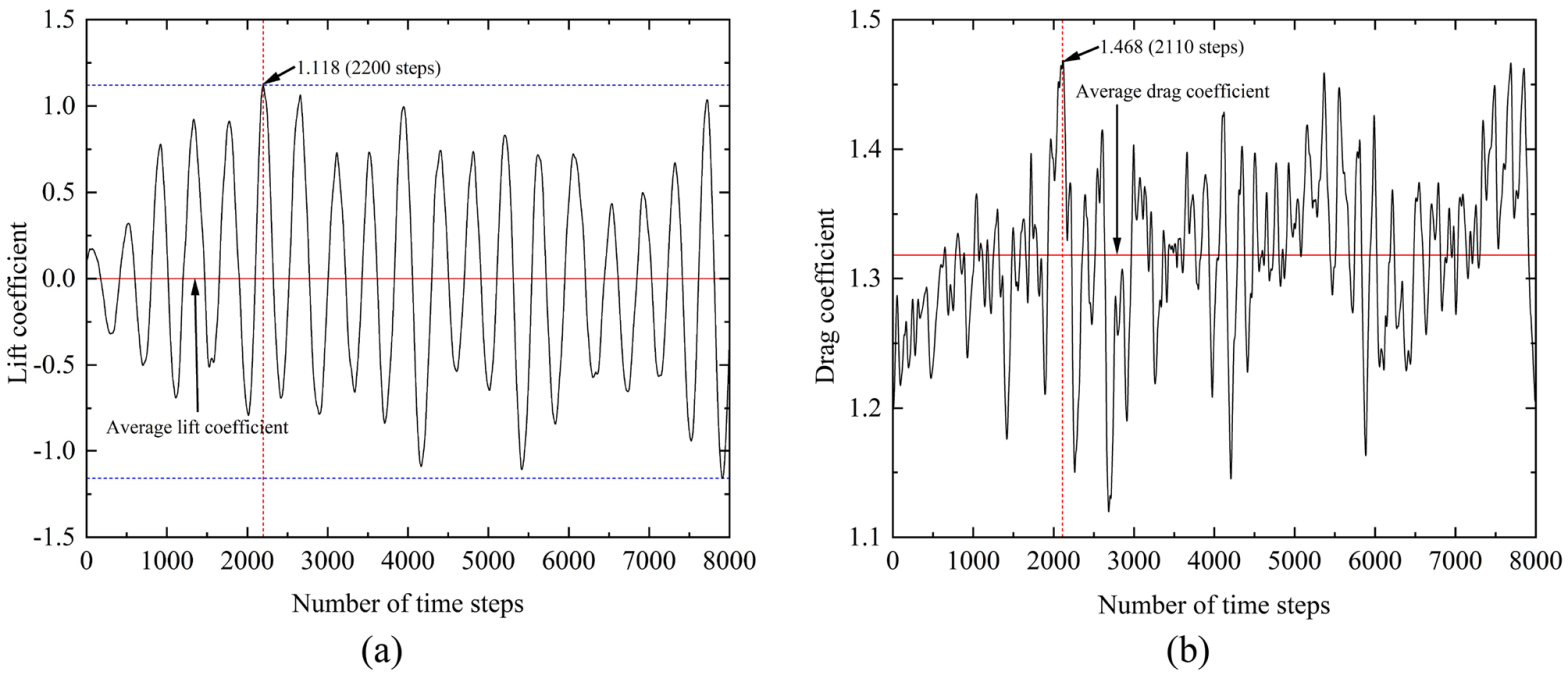
Aerodynamic coefficients time history: (**a**) Lift coefficients; (**b**) Drag coefficients.

Figure 6 shows the time history of lift coefficients and drag coefficients of the chamfered square cylinder in a time step of 8000. It can be seen from the Fig. 6 that the lift time-history curve of the chamfered cylinder is smooth and uniform, while the drag time-history changes greatly and the distribution is uneven. The maximum lift coefficient of the structure appears at 2200 steps, reaching 1.118. The maximum drag coefficient is 1.468, which occurs at 2110 steps. In general, the average lift coefficient of chamfered columns is about 0, and the fluctuating lift coefficient is 0.649. The average drag coefficient is 1.318.

**Figure 7:**
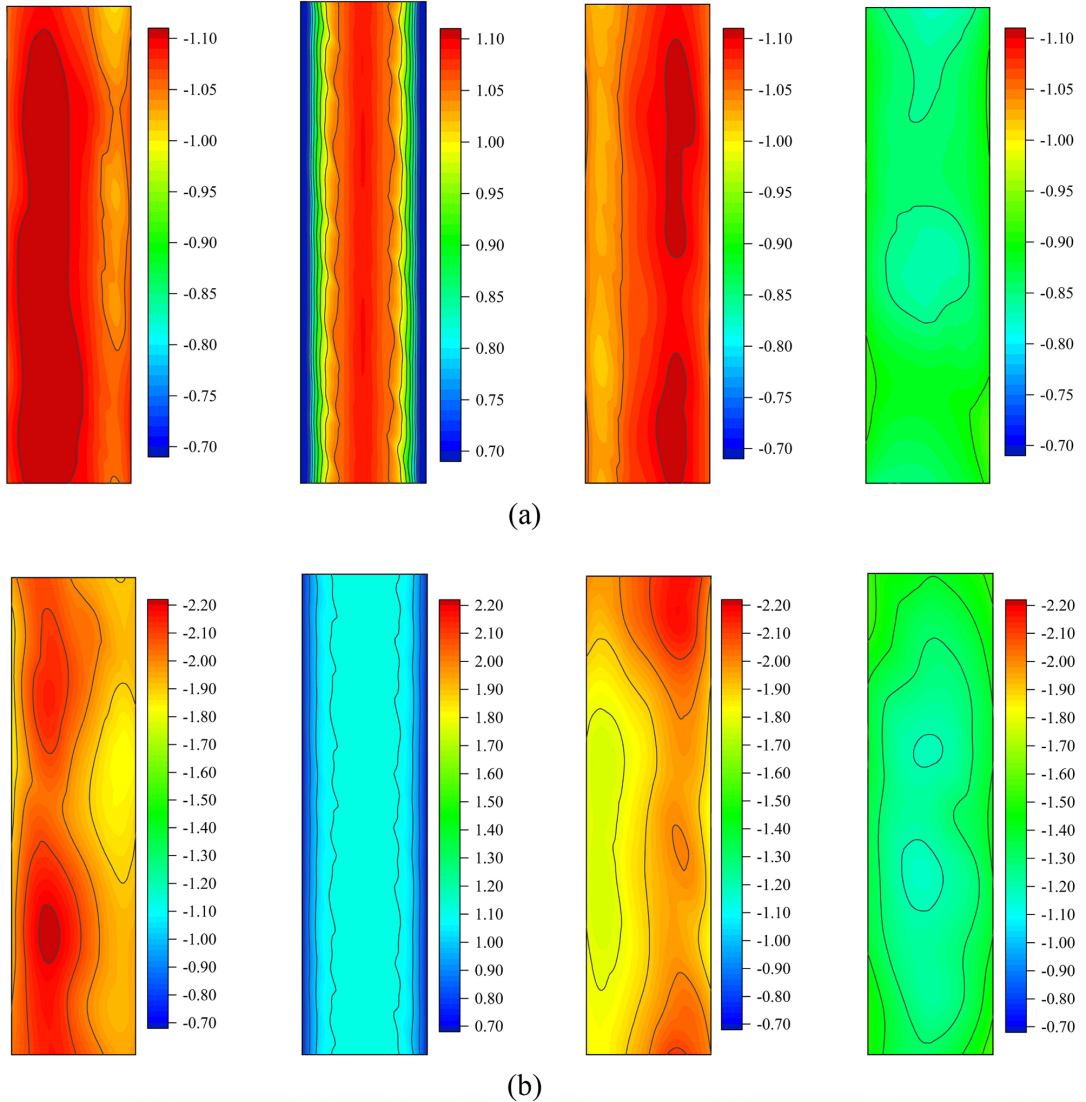
Wind pressure coefficients distribution on the isolated chamfered square cylinder: (**a**) Average wind pressure coefficients; (**b**) Extreme wind pressure coefficients.

Figure 7 shows the distribution results of the average and extreme wind pressure coefficients of the four facades of the isolated chamfered square cylinder (from left to right is the left facade, windward facade, right facade, and leeward facade). The wind pressure distribution has great symmetry. Most of the flank facades wind pressure coefficients (left and right facades) are greater than those of the windward and leeward sides, and the wind pressure coefficient of the left and right facades is almost the same, and the distribution is symmetrical. The low wind pressure area of the flank facade is located on the windward side (near the windward facade), and the high wind pressure area is located on the leeward side. The windward side of the cylinder shows positive pressure, and the wind pressure distribution is symmetric along the center axis of the calculation domain. The maximum value of the average and extreme wind pressure coefficients are -1.09 and -1.12 respectively, with a difference of only 0.03, indicating that the wind pressure on the windward side changes little with time. Both flank and leeward facades are distributed with negative pressure. The leeward facade has the smallest average wind pressure coefficient and the maximum value is only − 0.93, but the corresponding extreme wind pressure coefficient is − 1.58, and the ratio of maximum average and extreme wind pressure coefficient is 1:1.7. Both the maximums of the average wind pressure coefficient and extreme wind pressure coefficient are located on the left facade, with values of − 1.13 and − 2.22 respectively. The average wind pressure coefficient on the windward facade changes most sharply, and the difference between the maximum and minimum values is 0.63, while the difference between the side and the leeward facades does not exceed 0.12.

### Aerodynamic interference effect

**Figure 8:**
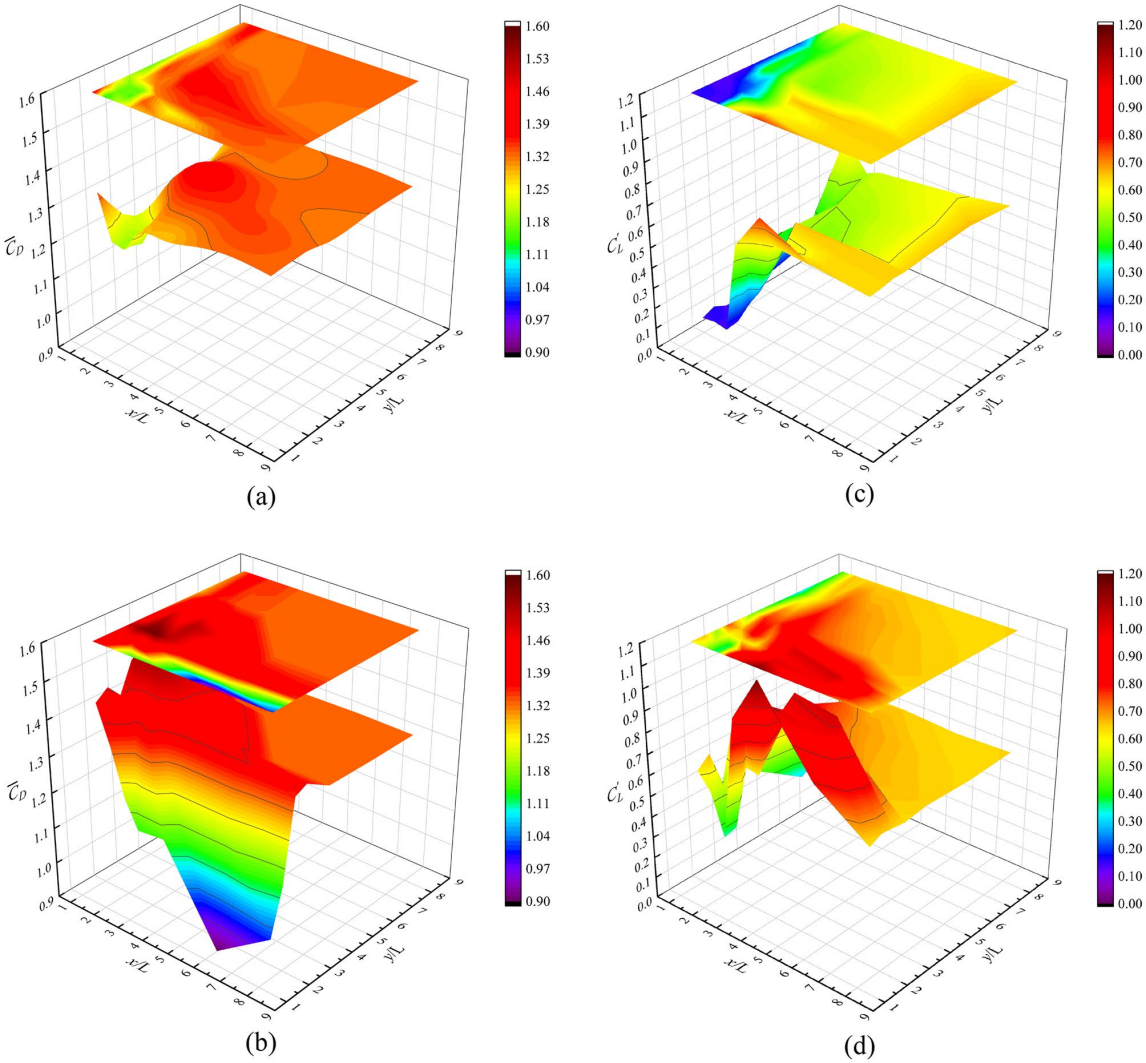
Distribution of aerodynamic coefficients of two square columns: (**a**) Average drag coefficients of interfering cylinder; (**b**) Average drag coefficients of principal cylinder; (**c**) Fluctuating lift coefficients of interfering cylinder; (**d**) Fluctuating lift coefficients of principal cylinder.

Figure 8 shows the aerodynamic coefficient distribution of the interfering square cylinder and the principal square cylinder at the interval from (1.5, 1.5) to (8.0, 8.0), where Fig. 8a,b shows the average drag coefficients, and Fig. 8c,d illustrates the fluctuating lift coefficients.

When the horizontal *y*/L distance is 1.5, the two square cylinders are similar to the tandem, and the drag coefficient is more sensitive to the along-wind flow field. At this time, $\overline{C}_{D}$ of the two square cylinders change in the opposite trend, and $\overline{C}_{D}$ of the interfering square cylinder gradually increases to the same as that of the isolated square cylinder, and $\overline{C}_{D}$ of the principal square cylinder gradually decreases to 0.901 due to the influence of the blocking effect. With the increase of *y*-axis spacing, the characteristics of the tandem gradually decrease, and $\overline{C}_{D}$ increases first and then decreases. When the coordinates are less than (2.5, 2.5), the interfering square cylinder shows a reduction effect in the partial coordinates, but the maximum value is 1.416 at the spacing of (1.5,1.5). $\overline{C}_{D}$ of the principal square cylinder shows an obvious amplification effect, in which $\overline{C}_{D}$ of the interfering square cylinder is slightly smaller than that of the isolated square cylinder at the spacing of (1.5,2.5). When the spacing is greater than (2.5,2.5), $\overline{C}_{D}$ of the interfering square cylinder is close to that of the isolated square cylinder, and $\overline{C}_{D}$ of the principal square cylinder is greater than that of the isolated square cylinder. When the spacing is greater than (6.0,4.0), $\overline{C}_{D}$ of the principal square cylinder and the interfering square cylinder is not affected by the spacing.

*C*^*’*^_*L*_ is mainly controlled by the cross-wind pressure, so it is more sensitive to *y*-axis spacing. When the spacing is less than (2.5,2.5), *C*^*’*^_*L*_ of the interfering square cylinder has a large difference from that of the principal square cylinder, and *C*^*’*^_*L*_ of the interfering square cylinder is less than 0.2, indicating that the cross-wind pressure fluctuation of the interfering square cylinder is small at this time. *C*^*’*^_*L*_ of the principal square cylinder is larger, and the maximum difference between *C*^*’*^_*L*_ of the principal square cylinder and that of the interfering square cylinder under the same working condition is 0.499. When the spacing is greater than (2.5,2.5), *C*^*’*^_*L*_ of the interfering square cylinder are all smaller than those of the isolated square cylinder, but* C*^*’*^_*L*_ of the principal square cylinder are greater than those of the isolated square cylinder. In general, *C*^*’*^_*L*_ of the interfering square cylinder does not exceed that of the isolated cylinder under different spacing, but *C*^*’*^_*L*_ of the principal square cylinder changes greatly. The minimum value of *C*^*’*^_*L*_ is 0.302, which appears at the spacing (1.5,6.0), and the maximum value is 1.151, which appears at the spacing (4.0,1.5). The difference between the two is 0.849.

### Non-Gaussian characteristics

At present, the evaluation criteria for non-Gaussian regions are mainly distinguished by structure types as shown in Table 2, where S represents skewness and K represents kurtosis. Based on the research results of Lou et al.^40^ and Han et al.^41^, the judgment standard of this paper is that the absolute value of skewness is greater than 0.2 and the kurtosis is greater than 3.5, or the absolute value of skewness is greater than 0.45, or the kurtosis is greater than 4.0.

**Table 2:** Judgment standard of non-Gaussian regions of different structure types.

Structure type	Judgment standard
Low-rise houses	|S|> 0.5, K > 3.5^38^
Large-span roofs	|S|> 0.5, K > 3.7^39^
Chamfered high-rise buildings	|S|> 0.2, K > 3.5^40^
High-rise buildings	|S|> 0.25, K > 3.2 or |S|> 0.45^41^ or K > 4.0
This paper	|S|> 0.2, K > 3.5 or |S|> 0.45 or K > 4.0

**Figure 9:**
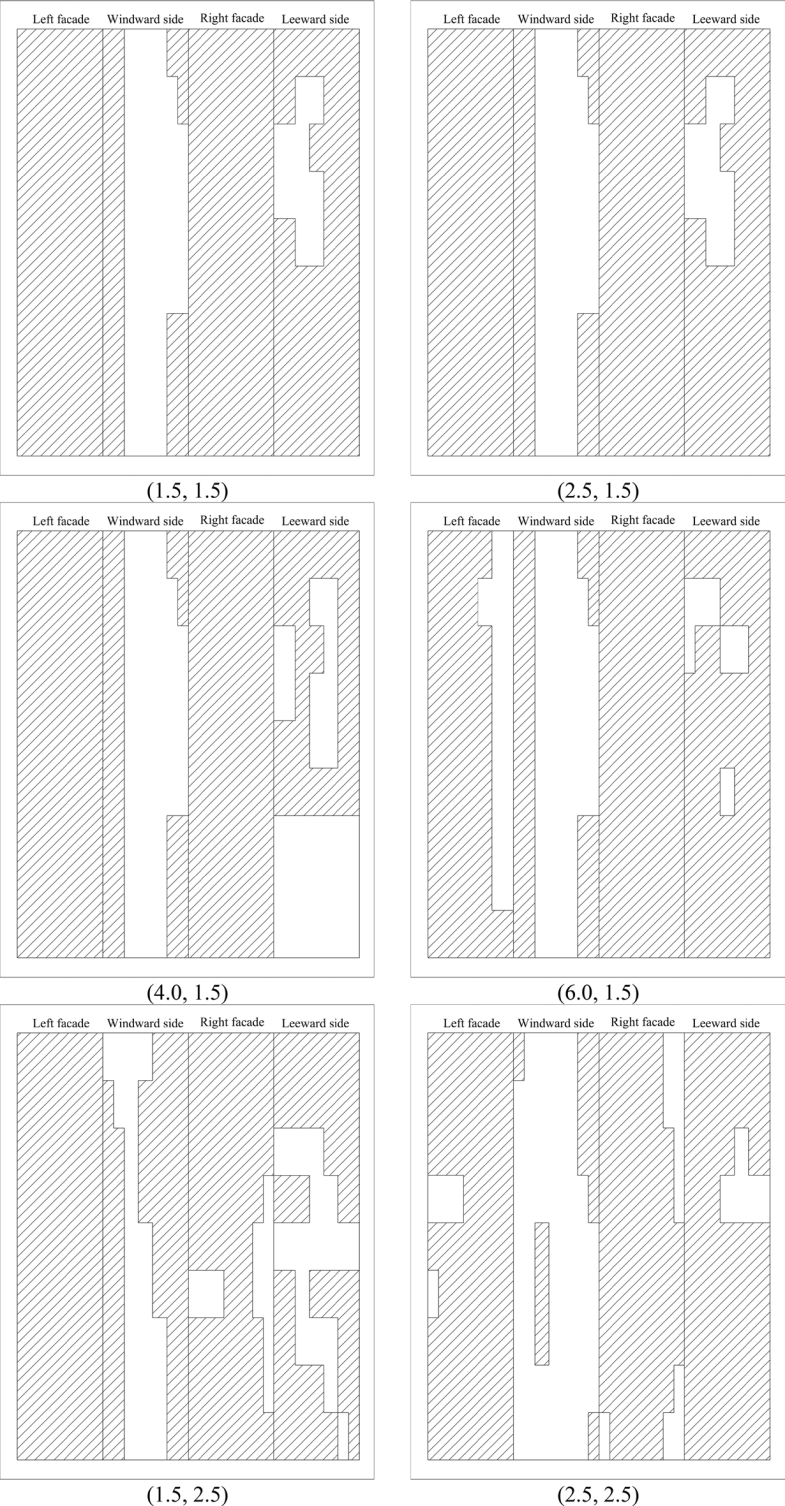

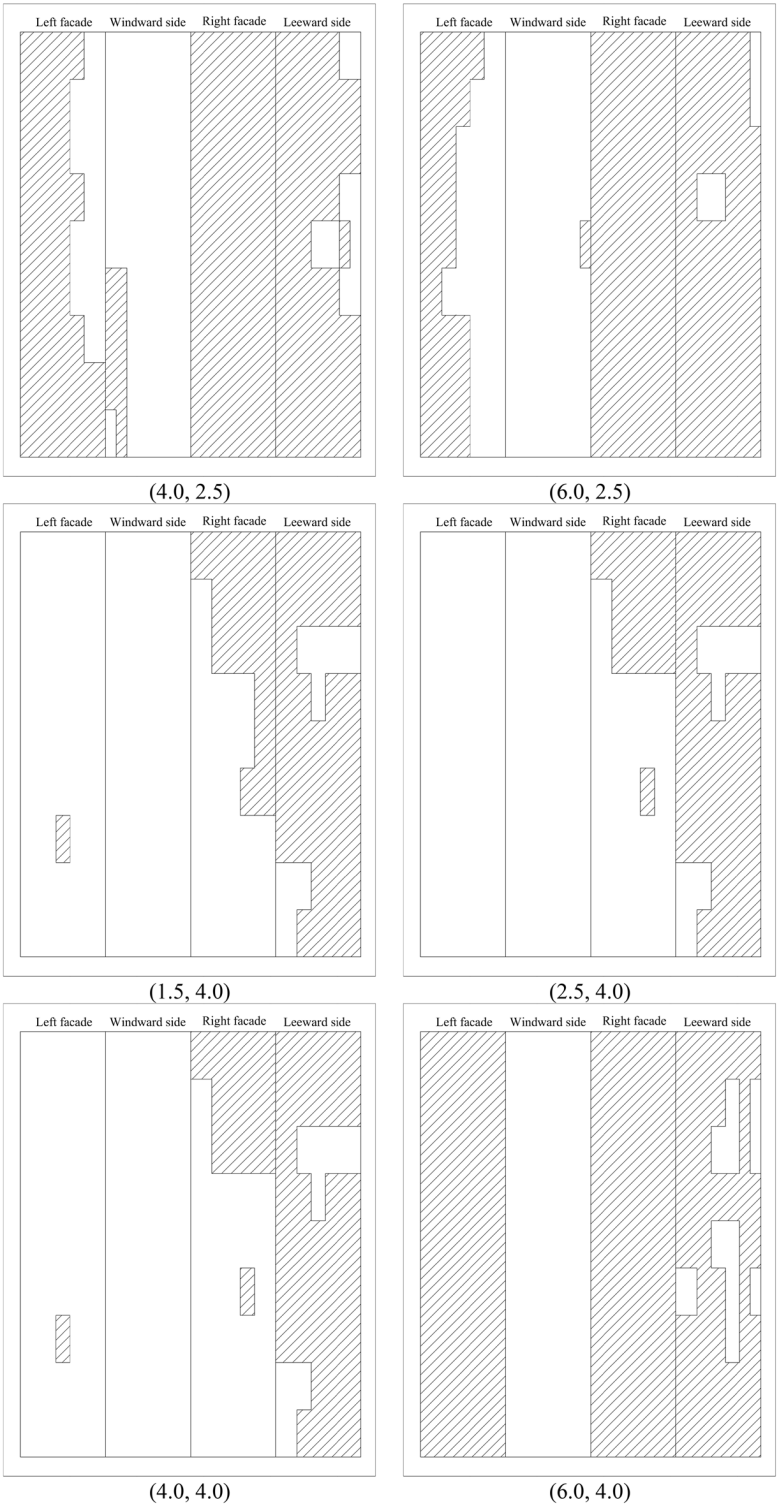

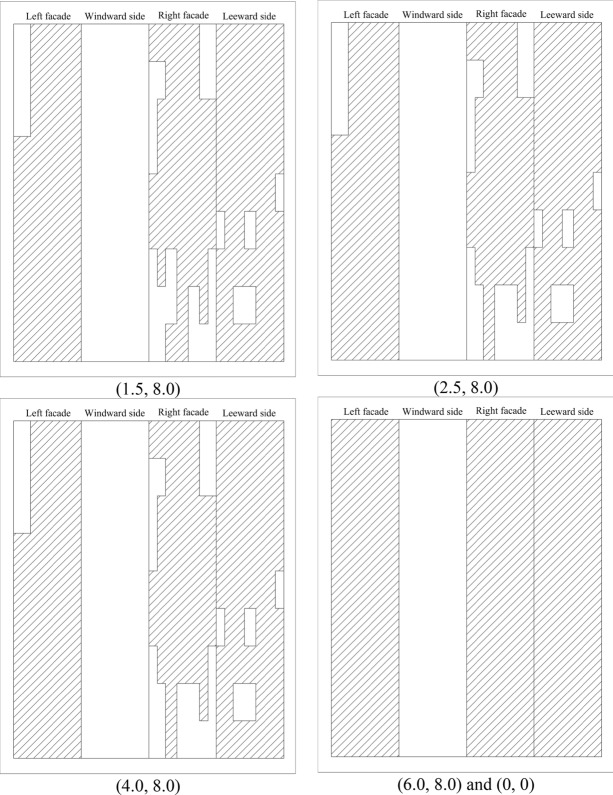
Non-Gaussian region distribution for typical working conditions (filled regions).

Based on the above criteria, the non-Gaussian regions of 100 working conditions are divided. Since the non-Gaussian regions of some spacings are identical, Fig. 9 only gives the results of the non-Gaussian regions of the parts of working conditions of the principal square cylinder. The blank part of Fig. 9 represents the Gaussian region of wind pressure, while the filled part shows the non-Gaussian region of wind pressure. The order of the four facades is left, windward, right and leeward facades. According to the vertical relationship with the wind direction, the windward and leeward facades are defined as the along-wind facades and the left and right facades are defined as the cross-wind facades.

For the along-wind facades of the square cylinder, the non-Gaussian distribution on the windward side is relatively symmetrical. When the cross-wind distance (*y*-axis) is 1.5L ~ 2.5L, the non-Gaussian region of wind pressure is mainly distributed at the chamfered edges on both sides of the facade, and the non-Gaussian region generally increases slowly. When the *y*-axis spacing is 4.0L ~ 8.0L, the non-Gaussian region on the windward facade disappears. If the non-Gaussian region of the windward facade is compared along the *x*-axis, when the distance along the *x*-axis is 1.5L ~ 8.0L, the non-Gaussian region does not change with the distance except when *y* = 2.5L. The leeward facade of the principal square cylinder is a non-Gaussian region, and the distribution of the non-Gaussian region is almost unaffected by the spacing. However, there is a Gaussian region at the bottom of the facade at the distance (4.0,1.5), which is different from the regularity of other facades. The kurtosis and skewness of the facade under this spacing are close to the limit value specified in the regional division, so it is Gaussian. In addition, the non-Gaussian area of the remaining working conditions covers at least 68.06% of the total area.

The area of the Gaussian region on the cross-wind facades of the square cylinder varies greatly. When the cross-wind distance is less than 4L, the wind pressure on the surface of the square cylinder is non-Gaussian, and the non-Gaussian region accounts for at least 85.65% of the total area. When the coordinates are from (1.5,4.0) to (4.0,4.0), the non-Gaussian area of the square cylinder decreases abruptly, and the non-Gaussian area of the left facade only accounts for 1.85%, and the non-Gaussian area of the right facade is less than 50%. When *y* = 4.0L and *x*-axis spacing is greater than 4.0L, the area of the non-Gaussian region increases abruptly, and the cross-wind facades exhibit completely non-Gaussian characteristics.

In general, the non-Gaussian characteristics of the principal square cylinder are mainly controlled by the cross-wind direction. When the spacing is greater than (4.0,4.0), the non-Gaussian regional distribution of the principal square cylinder is the same as that of the isolated cylinder. Due to the complexity of the flow field at cutting corners of the cylinder, each working condition shows obvious non-Gaussian characteristics, which is consistent with the isolated condition.

### Wind pressure interference effect

The wind pressure interference effect of square cylinders is mainly determined by the average wind pressure coefficients and the extreme wind pressure coefficients. At present, the peak factor method is mainly used to estimate the extreme wind pressure coefficient of each measuring point^42^:

19 $$ \hat{C}_{p} = \overline{C}_{p} \pm g\sigma_{p} $$

where *σ*_*p*_ represents the standard deviation of the wind pressure time history; *g* is the peak factor.

As for the value of the peak factor, in the *Load Code for the Design of Building Structures* GB50009-2012^11^, assuming that the structure meets the Gaussian distribution, it is recommended that the value of the peak factor be 2.5, and the confidence rate can reach 99.38%. However, the surface wind pressure of the actual structure is difficult to meet the Gaussian distribution, and the value of the peak factor in the non-Gaussian region is much greater than 2.5, which leads to the extreme wind pressure coefficient calculated based on this peak factor being too small. Therefore, this paper considers the distribution of the non-Gaussian regions and adopts the observed extreme value method and the full probability iteration method to calculate the peak factor, and the confidence rate can reach 99.50%. In order to observe the interference effect of the facades more directly, the interference factor *IF* is calculated by Eq. (20):

20 $$ IF = \frac{{\hat{C}_{pi} }}{{\hat{C}_{ps} }} $$

where $\hat{C}_{pi}$ is the extreme wind pressure coefficient of the measuring point of the principal square cylinder, and $\hat{C}_{ps}$ is the extreme wind pressure coefficient of the measuring point of the isolated square cylinder.

**Figure 10:**
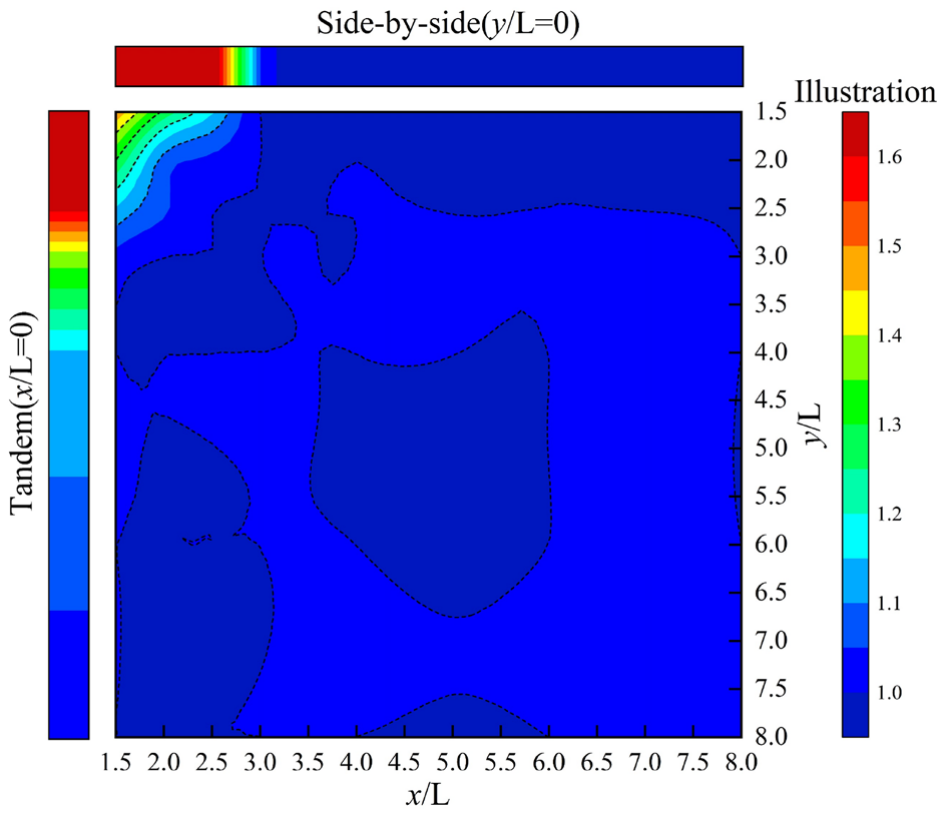
Along-wind interference effect of cylinder.

**Figure 11:**
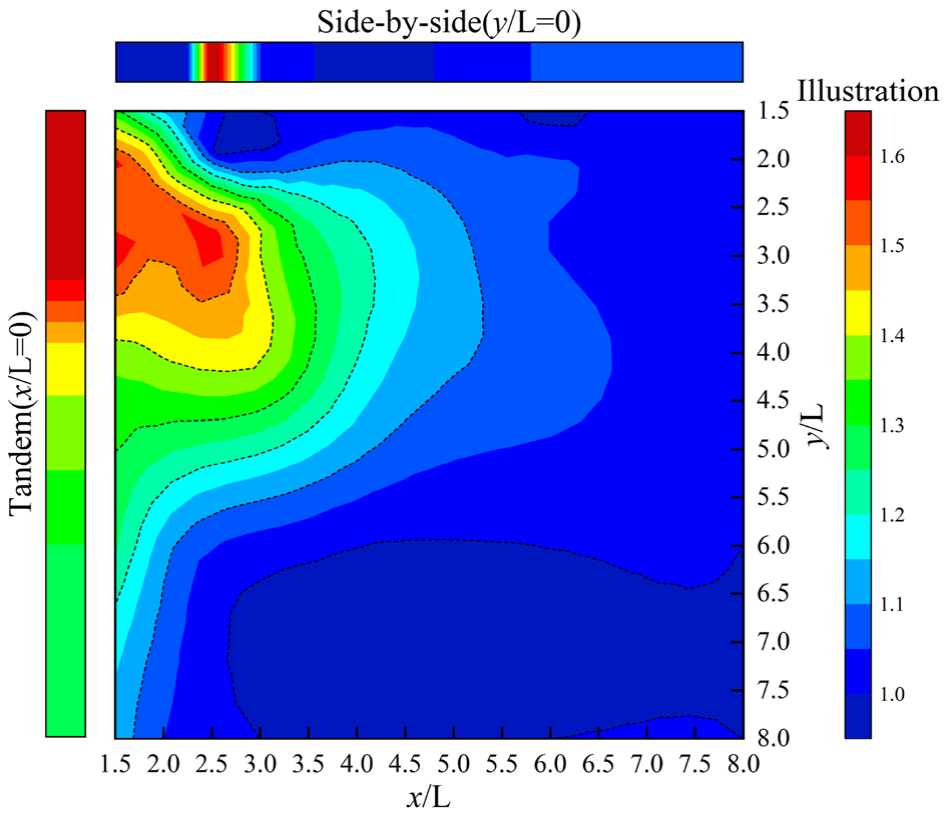
Across-wind interference effect of cylinder.

Figures 10 and 11 show the distribution of interference factors in the cross-wind and along-wind facades of the chamfered square cylinders in the 8.0L × 8.0L region based on Fig. 3. The maximum interference coefficients of cross-wind and windward facades are all located at the distance of 1.5L in side-by-side arrangements, and the corresponding distance coordinates are all (0.0,1.5), and the maximum interference factors are 2.58 and 2.38, respectively. When the coordinates are from (0.0,0.0) to (3.0,3.0), the along-wind interference effect of chamfered square cylinders is amplified, and the interference factor of chamfered square cylinders decreases with the increase of spacing (Fig. 10). The interference of the cross-wind facades is partly amplified, and the chamfered square cylinders are only reduced when they are arranged in tandem (Fig. 11). When the spacing is from (0.0,3.0) to (3.0,8.0), the chamfered square cylinder arrangement is similar to the side-by-side arrangement, and the interference factor of the along-wind facade is 1.30 ~ 1.00 (Fig. 10), and that of the cross-wind facade is 1.70 ~ 1.20 (Fig. 11). When the spacing is from (3.0,0.0) to (8.0,3.0), the working condition of the chamfered square cylinder can be compared to the tandem arrangement, and the along-wind interference factor of the chamfered square cylinder is 1.00 ~ 0.70 (Fig. 10), and the cross-wind facade interference factor is 1.30 ~ 1.00 (Fig. 11). When the distance is greater than (3.0,3.0), the interference effect on the along-wind facades disappears (Fig. 10), and the interference effect on the cross-wind facades is only greater than 1.00 at a small interval (Fig. 11).

In conclusion, the closer the spacing between the two cylinders, the more intense the interference effect, and the interference effect of the cross-wind facades is more intense and complex than that of the along-wind facades, and the interference factors of side-by-side arrangements are greater than these of the tandem arrangements. When the *y-*axis spacing is greater than 8.0, the cross-wind amplification effect of 20% still exists on the surface of the square cylinder, while when the *x*-axis spacing is greater than 3.0, the interference of the principal square cylinder tends to disappear.

## Conclusion

Based on the large eddy simulation method, this paper studies chamfered square cylinders with different spacing in the 8.0L × 8.0L region, calculates the aerodynamic distribution regularity of interfering and principal square cylinders, gives the non-Gaussian regional distribution of each facade of the principal square cylinder under various arrangements, and finally obtains the interference factor distribution of the along-wind and the cross-wind facades in the 8.0L × 8.0L region. The following conclusions are drawn:

1. The drag coefficient is more sensitive to the along-wind flow field, and the lift coefficient is mainly controlled by the cross-wind pressure. When the spacing is less than (2.5,2.5), the average drag coefficient of the perturbed square cylinder is less than that of the isolated square cylinder, and the fluctuating lift coefficient is less than 0.2. Due to the influence of the wake current of the interfering square cylinder, the average drag coefficient of the principal cylinder gradually decreases to 0.871, and the maximum difference between the fluctuating lift coefficients of the interfering square cylinder and the principal square cylinder is 0.594. When the spacing is greater than (2.5,2.5), the average drag coefficient of the interfering square cylinder is the same as that of the isolated square cylinder, and the fluctuating lift coefficient is slightly smaller than that of the isolated square cylinder. The average drag coefficient and fluctuating lift coefficient of the principal square cylinder show an obvious amplification effect.
2. All spacings of the cylinder chamfered corners present obvious non-Gaussian characteristics, and the distribution of non-Gaussian regions of each facade is not affected by the along-wind interval. When the cross-wind spacing is less than 2.5L, the non-Gaussian regions of the windward facade are mainly concentrated on both sides, while the leeward facade and both flanks are mainly non-Gaussian characteristics. When the spacing is greater than (4.0,4.0), the non-Gaussian region distribution of the principal square cylinder is consistent with that of the isolated square cylinder, the windward side shows the Gaussian characteristics, and the leeward facade and both flanks are the non-Gaussian regions.
3. The interference effect of chamfered square cylinders decreases with the increase of the spacing, the interference effect of the cross-wind facades is more severe than that of the along-wind facades, and the interference factors of side-by-side arrangements are greater than these of the tandem arrangements. When the side-by-side spacing reaches 8.0L, the interference effect of the cross-wind facades of the principal square cylinders is still more than 20%, and when the tandem spacing is greater than 3.0L, the interference effect of the principal square cylinder tends to disappear. In the 8.0L × 8.0L region, the maximum values of the cross-wind and along-wind interference effects are both located at (0,1.5), and the interference factors are 2.58 and 2.38, respectively.

## Data Availability

The datasets generated and/or analysed during the current study are not publicly available due to institutional confidentiality requirements but are available from the corresponding author on reasonable request.
